# ReliefSeq: A Gene-Wise Adaptive-K Nearest-Neighbor Feature Selection Tool for Finding Gene-Gene Interactions and Main Effects in mRNA-Seq Gene Expression Data

**DOI:** 10.1371/journal.pone.0081527

**Published:** 2013-12-10

**Authors:** Brett A. McKinney, Bill C. White, Diane E. Grill, Peter W. Li, Richard B. Kennedy, Gregory A. Poland, Ann L. Oberg

**Affiliations:** 1 Tandy School of Computer Science, Department of Mathematics, University of Tulsa, Tulsa, Oklahoma, United States of America; 2 Laureate Institute for Brain Research, Tulsa, Oklahoma, United States of America; 3 Division of Biomedical Statistics and Informatics, Department of Health Sciences Research, Mayo Clinic, Rochester, Minnesota, United States of America; 4 Mayo Clinic Vaccine Research Group, Mayo Clinic, Rochester, Minnesota, United States of America; 5 Department of Medicine, Mayo Clinic, Rochester, Minnesota, United States of America; 6 Program in Translational Immunovirology and Biodefense, Mayo Clinic, Rochester, Minnesota, United States of America; Institute of Biomedical Sciences, Taiwan

## Abstract

Relief-F is a nonparametric, nearest-neighbor machine learning method that has been successfully used to identify relevant variables that may interact in complex multivariate models to explain phenotypic variation. While several tools have been developed for assessing differential expression in sequence-based transcriptomics, the detection of statistical interactions between transcripts has received less attention in the area of RNA-seq analysis. We describe a new extension and assessment of Relief-F for feature selection in RNA-seq data. The ReliefSeq implementation adapts the number of nearest neighbors (k) for each gene to optimize the Relief-F test statistics (importance scores) for finding both main effects and interactions. We compare this gene-wise adaptive-k (gwak) Relief-F method with standard RNA-seq feature selection tools, such as DESeq and edgeR, and with the popular machine learning method Random Forests. We demonstrate performance on a panel of simulated data that have a range of distributional properties reflected in real mRNA-seq data including multiple transcripts with varying sizes of main effects and interaction effects. For simulated main effects, gwak-Relief-F feature selection performs comparably to standard tools DESeq and edgeR for ranking relevant transcripts. For gene-gene interactions, gwak-Relief-F outperforms all comparison methods at ranking relevant genes in all but the highest fold change/highest signal situations where it performs similarly. The gwak-Relief-F algorithm outperforms Random Forests for detecting relevant genes in all simulation experiments. In addition, Relief-F is comparable to the other methods based on computational time. We also apply ReliefSeq to an RNA-Seq study of smallpox vaccine to identify gene expression changes between vaccinia virus-stimulated and unstimulated samples. ReliefSeq is an attractive tool for inclusion in the suite of tools used for analysis of mRNA-Seq data; it has power to detect both main effects and interaction effects. Software Availability: http://insilico.utulsa.edu/ReliefSeq.php.

## Introduction

Gene expression data measured by next generation sequencing (*i.e.*, mRNA-seq data), has distributional properties that distinguish it from microarray gene expression data and genome-wide association study (GWAS) data. For example, not all genes are detected in all subjects, resulting in incomplete or zero-filled data matrices. In addition, the counts have been shown to have over dispersion that is consistent with the assumptions of the Negative Binomial distribution [1], [2]. Most analytical methods to date have focused on detecting per-gene differential expression [1], [3], [4], [5], [6]. In contrast, biological systems are regulated by nonlinear networks whose robustness to external and genetic perturbations may buffer or suppress the phenotypic effect of an individual transcript. For example, in model organisms there is evidence of the buffering effect of genes whose inactivation leads to increased phenotypic effect of other genes in the network [7], [8], [9], [10]. Changes in the activity of one gene product due to variation in the expression of another gene has been referred to as an epigenetic interaction [11], in contrast to statistical interactions between mutations, which are usually called epistatic interactions or epistasis. These effects may lead to non-additive statistical interactions in expression levels, where one gene mitigates the phenotypic effect of an interacting partner. Statistical interactions between genes may also be caused by differential co-expression across phenotype groups [12]. Thus, to understand how different sources of variation affect phenotypic outcomes, analytical techniques are needed that have the ability to detect single-gene effects as well as collective and interaction effects of multiple transcripts.

Relief-F is a nearest neighbor machine learning feature selection algorithm that is known for its ability to find relevant features that involve interactions [13], [14], [15]. Previous studies have shown that the quality of Relief-F estimates of conditionally independent features increases monotonically with the number of neighbor subjects (k) [16]. When the features are conditionally dependent, the feature quality is no longer monotonic, but rather reaches a peak and then decreases as the number of neighbors increases further. To be specific, conditional dependence refers here to the dependence between two genes conditional on the phenotype. It does not refer to the correlation between the two genes. Moreover, as k increases, Relief-F’s estimates of feature importance become more myopic, making the scores behave more like a univariate statistic [17]. Recent work has been done to find a uniform nearest-neighbor radius rather than specifying a number of nearest neighbors [18]. However, not only is it likely that different data sets require a different number of nearest neighbors, but each feature (*e.g.*, gene) within a data set also likely requires a different number of nearest neighbors for optimal attribute estimation. Thus, in this study, we propose a gene-wise adaptive-k (gwak) Relief-F algorithm for feature selection for RNA-seq data.

The primary goal of the current study is to describe an RNA-Seq analysis implementation of Relief-F where the number of nearest neighbors (k) is data driven and adapts to each gene. We call this implementation ReliefSeq and release it as an open-source tool for feature selection in RNA-Seq as well as GWAS data. The second, and essential, goal is to compare the power and assess the strengths and weaknesses of Relief-F with fixed and adaptive k, Random Forest, edgeR, and DESeq to detect main effects and interaction effects in RNA-Seq data. Random Forest is a machine learning classifier and feature selection algorithm with many robust properties, such as good prediction when many of the predictor variables are noise [19]. However, Random Forest has very limited power to detect gene-gene interactions in high dimensional data, as we have shown in GWAS data [15], [20] and others have confirmed in depth in Ref. [21]. These methods are a representative cross-section of statistical and machine learning methods that have been used either for RNA-Seq data in particular or high-dimensional data more generally to perform feature selection or dimensionality reduction. Finally, we apply ReliefSeq to a recently published data set that includes RNA-Seq data for virus-stimulated and unstimulated samples from subjects who received the smallpox vaccine [22].

## Methods

### Matrix Formulation of Relief-F Feature Selection

**Figure 1:**
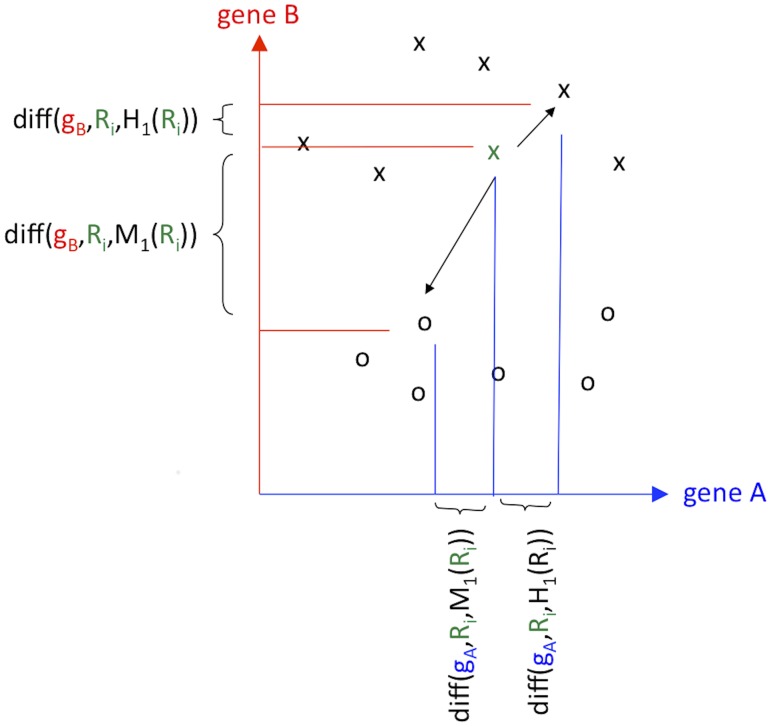
Two-dimensional (two gene) illustration of the gene-wise adaptive-k Relief-F algorithm. First, a subject Ri (green x in the figure) is selected and its nearest k hits and misses are found in the space of all genes. In the figure, k = 1 is shown as an example, and the nearest hit of Ri (nearest×symbol) and its nearest miss (nearest circle symbol) are indicated by vectors (arrows) in the full space of genes. To estimate the contribution of subject Ri to the ability of each gene to discriminate between cases and controls, we calculate the difference (diff) in the expression of the gene between Ri and its hits and between Ri and its misses. These hit and miss differences are illustrated as projections onto the gene A (blue) and gene B (red) axes. Subject Ri contributes a positive discrimination for gene B because the difference between the miss projection and Ri is greater than the difference between the hit projection and Ri. Subject Ri contributes nearly 0 to the importance of gene A because the miss and hit projected differences are almost the same. These estimates of the differential expression of each gene are averaged for each subject (the above steps are repeated for each subject Ri). The above Relief-F algorithm is repeated for a range of k, resulting in each gene having an array of scores corresponding to each k. The highest Relief-F score is used for each gene.

Relief-F is a nearest-neighbor-based machine learning feature selection algorithm that has the ability to identify features based on main effects or interactions. However, the ability to find these two different types of effects depends on the number of nearest neighbors. With a fixed number of nearest neighbors, Relief-F is better at finding one effect at the expense of the other. Thus, we introduce a new Relief-F importance weighting method that adapts the number of nearest neighbors to identify both types of effects. That is, the adaptive number of nearest neighbors is allowed to be different for each transcript in order to capture both main effects and interaction effects. To clarify how the new method differs from the original, we reformulate the original Relief-F weight for a transcript α as a difference of means in matrix form:
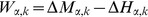
(1)where the quantities
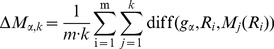
(2)and
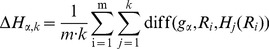
(3)are the mean deviations with respect to transcript α of the m subjects Ri from their k-nearest-neighbor (kNN) misses [Mj(Ri) in Eq. 2] and hits [Hj(Ri) in Eq. 3]. The set of misses for a subject Ri, 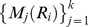, is the set of k subjects that are nearest to Ri but are in a different phenotype class than Ri. Likewise, the set of hits, 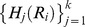, is the set of k subjects that are nearest to Ri while being in the same phenotype class as subject Ri. The diff function computes the difference in the abundance of transcript α between two subjects Ri and Rj:
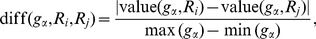
(4)where value(gα,Ri) returns the abundance of transcript or gene gα for subject Ri, and max and min for transcript or gene gα are calculated across all m subjects. The diff function is based on a single transcript α; however, it is also used in a Manhattan metric to determine the kNN hits and misses in the full space of transcripts. Thus, although the difference in the numerator of Eq. (1) is computed for a single transcript at a time, the mean deviations [Eqs. (2) and (3)] use the distance matrix from the entire space of transcripts to determine the kNN hits and misses if kH+kM<m. See Figure 1 for a visual explanation of how the algorithm works.

### ReliefSeq: Gene-wise Adaptive-k Relief-F tool for RNA-Seq Feature Selection

**Figure 2:**
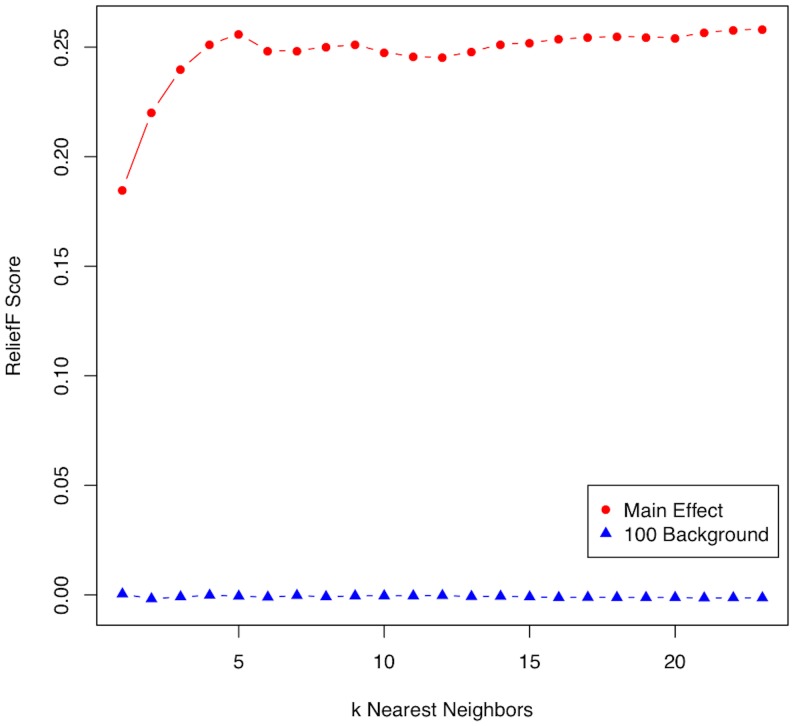
Illustration of the effect of the number of nearest neighbors (k) on the Relief-F score for a main effect transcript (red circles) from one of the simulated data sets. The vertical axis is the Relief-F importance score (Equation (1)); the horizontal axis is the number of nearest neighbors, k. For main effects, a larger k is suggested because Relief-F converges to a univariate attribute estimator (myopic). For comparison, the average scores for 100 random null transcripts (blue triangles) in the data set are plotted versus k. Regardless of k, the Relief-F scores remain low for noise genes.

**Figure 3:**
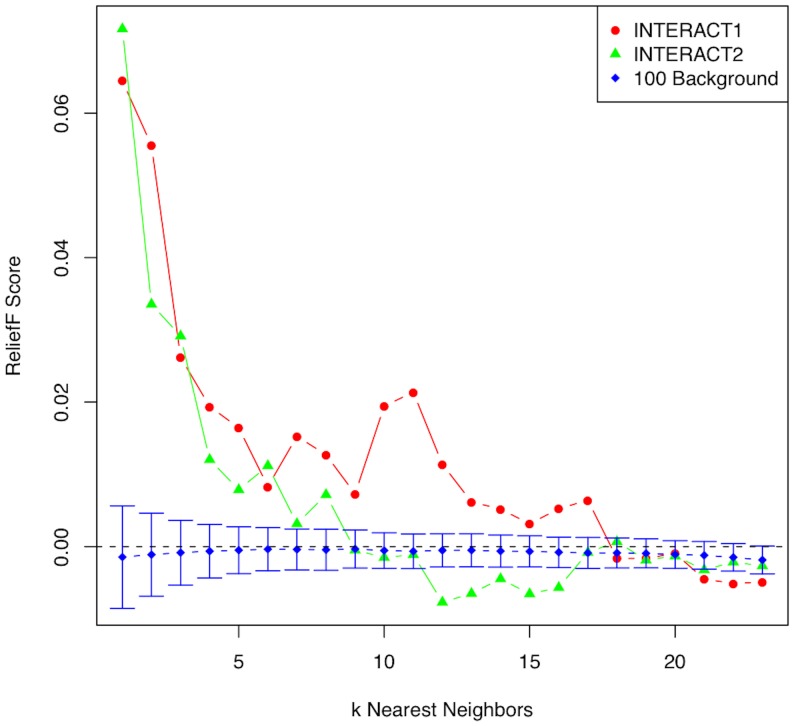
Illustration of the effect of the number of nearest neighbors (k) on the Relief-F score for two interacting transcripts (red circles and green triangles) from one of the simulated data sets. A lower k is optimal for interacting transcripts. For larger k, Relief-F converges to a univariate attribute estimator (myopic) and the scores of the interacting transcripts tend toward zero because they have no main effect. For comparison, the average scores and 5^th^ and 95^th^ percentiles for 100 random null transcripts (blue diamonds) in the data set are plotted versus k. The Relief-F scores for noise genes are low and relatively unaffected by the value of k.

In the original Relief-F algorithm, the number of nearest neighbors, *k*, from the hit and miss subjects is a parameter that must be specified. However, different types of effects in a data set warrant different values of k. To illustrate the rationale behind the selection of k, we plot the Relief-F score versus k for a simulated main effect along with the average score of 100 background or null transcripts (Figure 2). For main effects, a larger k is needed because the Relief-F algorithm becomes myopic and performs like a univariate attribute/feature estimator. For noise or background genes, the scores remain low and show little variation with respect to k. In contrast to main effects, the detection of interactions of the type described in the introduction requires a more moderate value of k (Figure 3). We illustrate this effect of k on Relief-F scores for interacting genes by plotting the Relief-F scores for two interacting transcripts without individual main effects along with the average score of 100 null transcripts in Figure 3. The scores of the two functional transcripts have a maximum for small k, and then for larger k the scores decay toward zero as Relief-F becomes more myopic. The Relief-F scores of noise variants again remain near zero regardless of the value of k.

Because we expect a mixture of main effects and interactions in a given data set, we expect a mixture of k values will be required for different transcripts. To address the gene-wise optimal number of nearest neighbors, we sweep k from 1 to k_max_ to create a matrix of Relief-F scores in Eq. 1. Note that the matrices in Eqs. (1–3) have row indices (α) for transcripts and column indices (k) for the number of nearest neighbors. We find the k for each transcript row that maximizes the weight in Eq. 1:
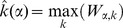
(5)and the ReliefSeq weight for transcript α becomes
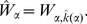
(6)


### Availability of ReliefSeq Code

All methods used in this study are available as C++ source code and executable at http://insilico.utulsa.edu/ReliefSeq. The data are assumed to be counts normalized for between-lane differences prior to analysis. No scaling is performed prior to applying these analyses. Sample input files are provided with the source code.

### Univariate Comparison Methods: edgeR and DESeq

edgeR and DESeq are analysis tools that can be used to perform differential expression in mRNA Seq data [1], [3], [23]. Both assume over-dispersion is present in data from independent biological replicates and that the variance increases as a quadratic function of the mean. This assumption has been empirically assessed and found to be reasonable [2]. In addition, both tools allow the dispersion parameter to vary between transcripts. The edgeR tool uses an approximate Empirical Bayes strategy akin to that used in the popular LIMMA package [24] for gene expression microarray data to estimate the dispersion parameter, thus sharing information across transcripts in the process. Default settings were used for function parameters. The DESeq tool implements a local regression to empirically estimate the dispersion parameter, thus also sharing information across transcripts in the process. Default DESeq settings were used.

### Machine Learning Comparison Method: Random Forest

Random forest is an ensemble machine learning classifier based on bagging (bootstrap aggregation) of classification or regression trees combined with random selection of variables for each node split [19]. Multiple trees are learned on bootstrap samples of the data and then either the trees are averaged for regression or polled for a majority vote in the case of classification. The algorithm has two main parameters: the number of variables (mtry) selected randomly for each node split and the number of trees (ntrees) in each forest. Previous studies have shown that default parameters are typically effective [25], but we test this for simulated RNA-seq data. We use three different versions of Random Forest: the randomForest() version in R that uses the default mtry (the square root of the number of predictor variables), the tuneRF() version in R with optimization of mtry through cross validation, and Random Jungle, which is also part of our evaporative cooling (EC) tool for GWAS [26].

### Simulations

Data were simulated in order to assess the operating characteristics of the feature selection strategies in a situation where truth is known. Null simulations containing no true differential expression were generated in order to understand control over type I error. In order to understand power, data sets were simulated containing either main effects or buffering interaction effects between two transcripts. One thousand simulated replicate data sets were created for each main effect scenario described below and one hundred replicates for the interaction simulations. No differences in library size (total number of counts per sequencing lane) were built into the simulations since these should be normalized out in the preprocessing. Since the library sizes were approximately constant, no normalization was performed when analyzing the simulated data.

#### Distribution of gene expression

Count data for each gene were assumed to follow a Negative Binomial distribution with mean E(y) = µ and Variance(y) = µ+µ^2^/θ where θ is the dispersion parameter in keeping with previous literature [1], [2]. An existing mRNA Seq data set of 23 subjects [2], [27] was used to create the population mean vector by calculating the sample mean for each transcript which ranged from 0.04 to approximately 100,000. The moderated dispersion parameters as estimated by edgeR on the same dataset were used in the population dispersion parameter vector and ranged from 1.5 to 15,000. 16,920 genes were simulated. For each combination of parameters, 1000 replicate data sets were simulated for each model unless otherwise specified in the results. Null simulations (*i.e.*, no study group differences) were created by using these values to simulate random negative binomial numbers for n = 48 subjects.

#### Main effect simulations

For main effect simulations, two study groups were created of n = 24 each by adding fold changes of FC = 0, 0.25, 0.5, 0.75, 1 on the log2 scale (1, 1.18, 1.41, 1.68, 2 on the raw scale) to the population mean for 96 main effect transcripts in a background of null genes for a total of 16,920 transcripts. In order to assess performance for transcripts across the range of distributional characteristics, these 96 transcripts had mean counts (µ) from 1.5 to 1,500. 1000 simulated data replicates were created, each containing 96 main effect transcripts. Thus, these 96 genes should have ranks between 1 to 96, and the null genes should have ranks from 97 to 16,920.

#### Gene-gene interaction simulations

**Figure 4:**
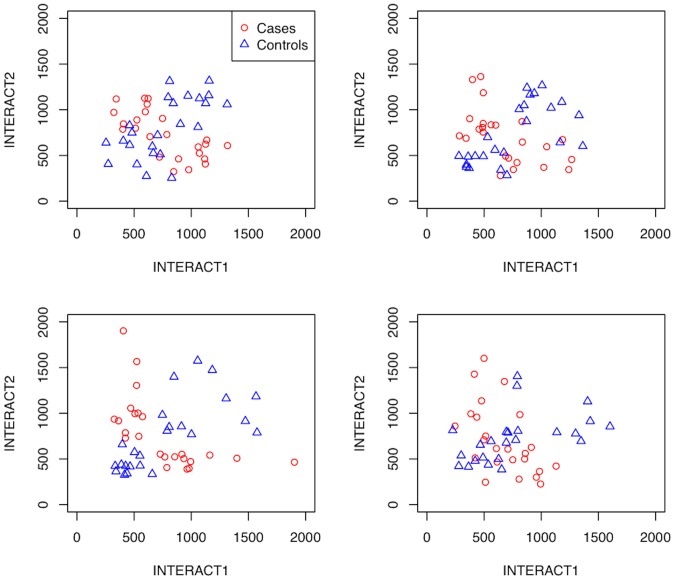
Example scatter plots of simulated data for two interacting transcripts. Each scatter plot is a replicate of the same XOR simulated model. Cases and controls are labeled in the 2-dimensional space of the two interacting transcripts (labeled INTERACT1 and INTERACT2). The simulated data includes approximately 16,000 null genes, but only the two interacting genes are shown. The XOR model is designed so that neither interacting gene by itself has a statistically significant differential expression between cases and controls (no main effect).

For interaction simulations, we constructed exclusive OR operator (XOR) models with negative binomial distributions for the pair of interacting transcripts. The scatter plots of subjects in Figure 4 illustrate a pair of interacting transcripts for different negative binomial parameters. Each gene has no effect by itself (*i.e.*, no marginal main effect) on the case/control status, but together they predict the outcome status. The XOR interaction model is one of the most challenging for statistical methods to identify, and it is motivated by environmental and genetic buffering and epistatic effects. In the XOR model, when both of the functional transcripts have a very high or very low abundance, the regulatory network system buffers the phenotypic effect, resulting in an individual being unaffected. However, if the abundance of one of the functional genes is high while the other is below detection by the feedback mechanisms of the epigenetic or regulatory network, then there is a threshold effect that leads to a subject being an affected case with varying probability or penetrance.

To simulate the two-class (case/control) interaction between two negative binomial variable genes, we divide the total sample population of 48 into two groups: 24 low expressed and 24 high expressed for both genes. For the low expression group, we generate counts based on the independent negative binomial distribution. For a given model, the low expression means of these genes vary from 200 to 10,000 and theta varies from 9.96 to 15.34. These values represent the baseline levels of a gene with a low expression. For the high expression group, we generate gene expression values for the other half of the subjects by adding a fold change of 2 to the low expression population mean for both genes. The high expression group will, on average, have twice the expression of the low expression group for the two genes. For the two genes, the half of subjects with a shifted mean are categorized as high expressers for that transcript, and the other half are categorized as low expressers. At this stage in the simulation we have not assigned a phenotype or class label to the subjects, so there is not yet an interaction or dependence between the genes conditioned on the phenotype.

Next we randomly permute the subjects of one of the pair of genes such that half of the subjects with high levels for the permuted gene are matched with high-level subjects for the other gene, and consequently half of subjects with high levels for the permuted gene are randomly matched with subjects with low levels for the other gene. Finally, we use the XOR function between the two genes to assign the case/control status of the subjects. Specifically, when one or the other gene is over expressed, but not both, for a given subject we assign this subject to a case status. Subjects for which both genes are over or under expressed, we assign to a control status. This process creates a pure interaction of the type described in the introduction between two negative binomial genes, and ensures that neither gene has a main effect except possibly by chance. A variable number of independent negative binomial background (noise) genes are appended to the interacting genes for each replicate simulated data set. The number of background (decoy) genes ranges from 100 to 12,800.

A final set of separate simulations was performed in order to understand the effect of the number of null features on the ability to detect the interactions. The simulated datasets were designed to have different numbers of null genes for each data set (100, 200, 400, 800, 1600, 3200, 6400 and 12800 total genes). For each combination of negative binomial parameters and number of null genes, we generated n = 100 replicate data sets. Each simulated data set had 48 balanced subjects and one pair of functionally interacting genes.

### Method Comparison Metrics

In order to assess the operating characteristics of the feature selection strategies, the rank of each transcript was calculated for each analysis strategy in each simulation. The average rank of each functional gene was computed across the n = 1000 replicate simulations of each main effect model. For interaction models, we used n = 100 replicates for a given model and we averaged the worst rank of the pair of interacting genes across the replicates. Using the worst rank of the pair instead of averaging the ranks of the pair avoids the potential of one transcript having a very good rank by chance and biasing the performance of the method. For clarity, the variability is not shown in the plots but is included in Tables S1–S3.

## Results

### Main Effect Simulation Results

**Figure 5:**
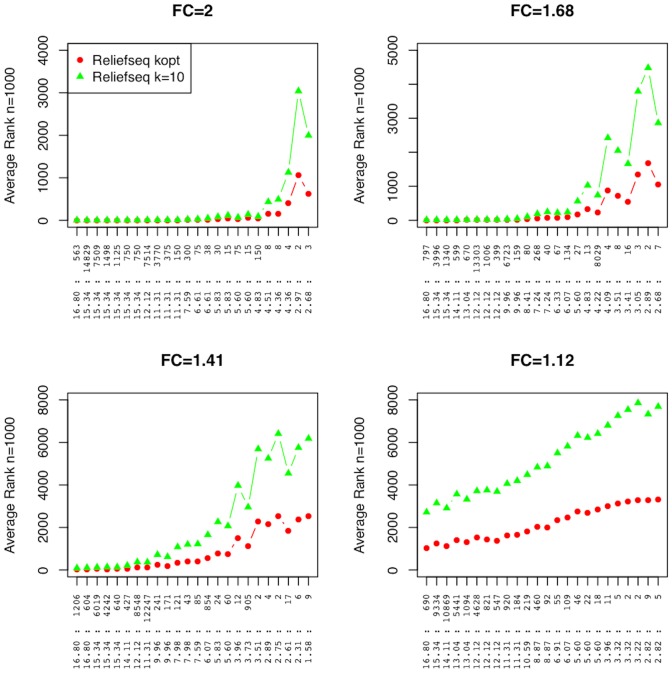
Main effect simulation results comparing gene-wise adaptive-k ReliefSeq with standard Relief-F (k = 10). Average ranks of 96 main effects of different size for ReliefSeq with gene-wise adaptive k compared with standard Relief-F with k = 10 nearest neighbors. The 96 main effect genes are simulated in each data set with a background of null genes for a total of 16,920 genes. The 96 main effect genes are split into four panels based on fold change (FC) labeled on the raw scale. The horizontal axis of each plot is labeled by the combination of negative binomial parameters (θ:µ) of the main effect gene. Genes toward the right of a panel have smaller effect size, making them more difficult to detect. Each plot point is the average rank across n = 1000 replicate simulations.

The standard fixed-k Relief-F uses a relatively large, k = 10, number of neighbors, making it best for estimating main effects. We compare gene-wise adaptive-k ReliefSeq with k = 10 Relief-F for a variety of negative binomial main effect models (Figure 5). Discussed in more detail in the Methods, each simulated data set contains 96 main effect transcripts together with 16,824 null genes for a total of 16,920 genes. Effect sizes in each panel are ordered strongest on the left and weakest on the right. The two methods perform similarly for large effect sizes, but gwak-ReliefSeq yields better ranks for weaker main effects. Both strategies perform similarly for fold changes of 2 and 1.68. Optimized-k ReliefSeq has lower mean rank in general for fold changes of 1.41. Both versions of Relief-F have difficulty detecting fold changes of 1.12. Variability in the rankings increases with the mean rank, but is within ±11 or less of the average rank for all but one gene with rank less than 100 for ReliefSeq with optimized k (Table S1). The variability in ranks is larger for Relief-F with fixed k and reaches ±20 ranks for several genes.

**Figure 6:**
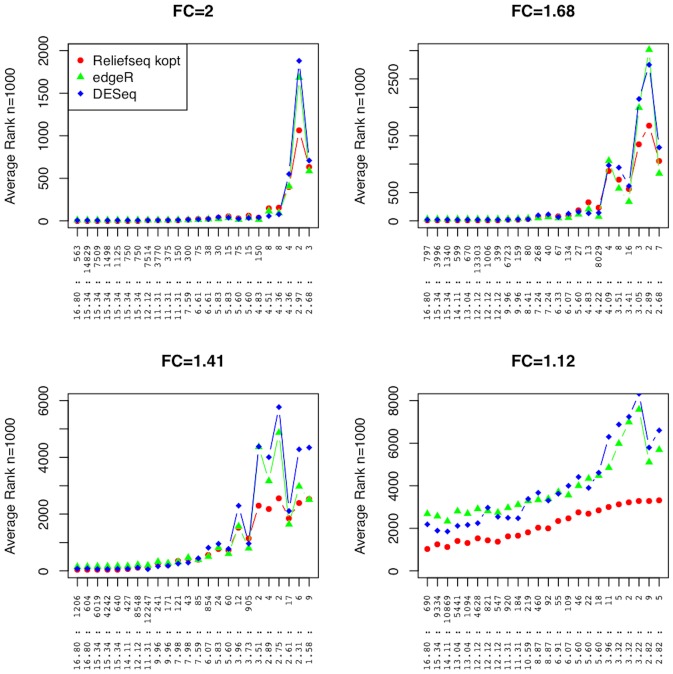
Main effect simulation results comparing gene-wise adaptive-k ReliefSeq, edgeR, and DESeq. Average ranks of the 96 main effect genes simulated in each data set with a total of 16,920 genes for Relief-F with optimized k nearest neighbors, edgeR, and DESeq. The results are split into four panels based on fold change (FC) on the raw scale. The horizontal axis shows the 96 simulated main effect genes, ordered left to right from high to low main effect strength, and labeled by the combination of negative binomial parameters (θ:µ) of the main effect gene. Each point is the average rank across n = 1000 replicate simulations (vertical axis).

Two of the established univariate methods for identifying differentially expressed genes in RNA-Seq data are edgeR and DESeq. Thus, we compare the rankings by gwak-ReliefSeq, edgeR, and DESeq in the simulated main effect data sets (Figure 6). The methods perform similarly and the ability to detect real signal deteriorates for all methods as the fold change decreases and as the mean µ decreases. The mean rank for a fold change of 2 begins to increase as the mean counts µ is at 30 or fewer counts. For a fold change of 1.68, the mean rank begins to increase once µ is less than approximately 500. All analytical methods have difficulties detecting fold changes of 1.12 where all of the average rankings are well above 1000, regardless of µ or θ.

**Figure 7:**
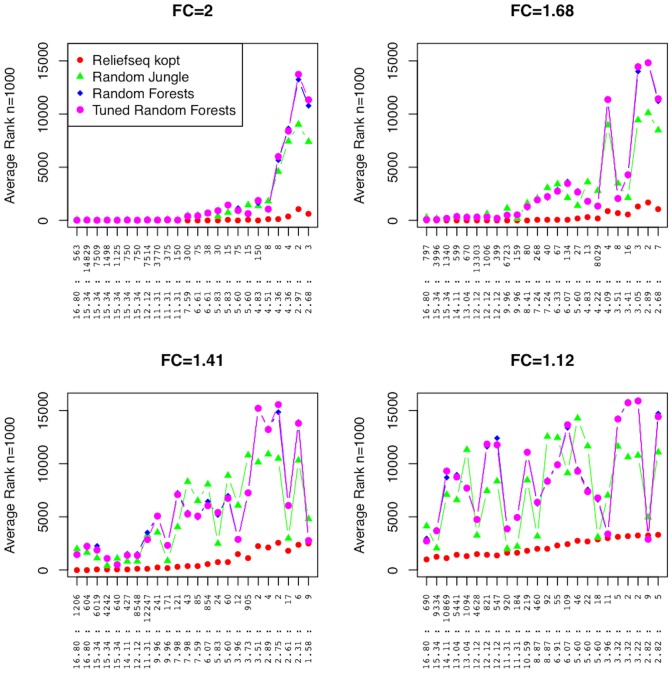
Main effect simulation results comparing gene-wise adaptive-k ReliefSeq and multiple Random Forest implementations. Same panels and axes as in Figs. 5 and 6, but Relief-F with optimized k nearest neighbors is compared with Random Jungle, Random Forest (R implementation), and tuned Random Forest with optimized mtry. Average ranks of the 96 main effect genes simulated in each data set together with null genes for a total of 16,920 genes. The 96 genes are split into four panels based on fold change (FC). The horizontal axis of each plot is labeled by the combination of negative binomial parameters (θ:µ) of the main effect gene. Each point is the average rank across n = 1000 replicate simulations.

Random Forest is one of the most widely used methods for classification and feature importance ranking from the field of machine learning. Thus, our last comparison for main effects is between ReliefSeq and multiple implementations of Random Forest (Figure 7). For large fold changes and large effect sizes, Random Forest rankings are competitive with the other methods compared above. Random Forest average rankings begin to increase in the same regions as DESeq and edgeR, but they increase more quickly; Random Forest is essentially unable to detect fold changes of 1.41 and 1.12. Variability in rankings again increases with the mean rank, and is at the level of ±30 ranks for genes with average rank in the 80′s for Random Jungle and tuned Random Forest, and ±25 ranks for a gene ranked in the 50′s for Random Forest (Table S3).

### Interaction Simulation Results

**Figure 8:**
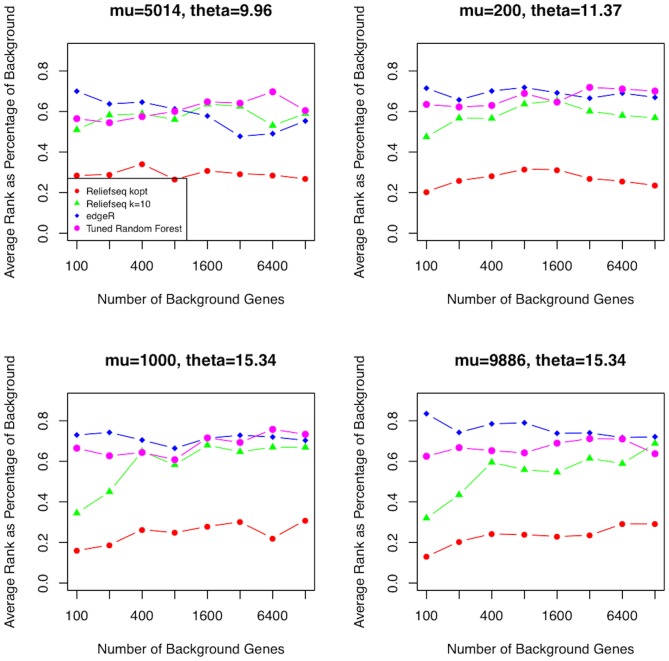
Interaction simulation comparison of feature ranking methods for different numbers of null genes. Comparison of optimized-k Relief-F, standard Relief-F using k = 10 nearest neighbors, edgeR, and tuned Random Forest for detecting an interaction between two genes amongst various numbers of null genes. The panels are sorted in order of increasing negative binomial parameter θ. Each point is the average of the worst ranking gene of the two simulated interacting genes across n = 100 replicate simulations and then divided by the total number of simulated genes. The number of null or background genes increases from 100 to 12,800 total genes, plotted on the log2 scale. Each simulation contains one pure interaction (no main effects) XOR model between two negative binomial genes.

One of the advantages of the nearest neighbor approach of Relief-F is that nearest hits and misses are determined in the space of all transcripts, which provides potential interaction information from other transcripts when ranking the importance of a given transcript. In contrast, myopic or univariate methods typically ignore information from other transcripts. In Figure 8 we compare the ability of multiple methods to detect epistatic interactions of the form described in the introduction and methods for simulated datasets with various numbers of null genes. ReliefSeq consistently gives better rankings than the other methods (edgeR, tune Random Forest, and k = 10 Relief-F) for this difficult XOR interaction model. The rankings are lower for larger µ, and ReliefSeq consistently ranks the interacting genes approximately 10% higher than Relief-F with fixed k, tuned Random Forest and edgeR. The other methods rank the interactions consistently at the 40–55^th^ percentile of features, which is not surprising given these methods were not designed to detect interactions of the form studied here. Interestingly, when viewed as a percent rather than absolute ranking, all analytical methods rank the interacting genes at a consistent position in the list, regardless of the number of null genes in the dataset. This consistency is not surprising for univariate methods, but ReliefSeq’s nearest hit and miss calculations performed in the whole transcript space have the potential to become less accurate when irrelevant genes are included. The relatively flat rankings as a function of number of background genes suggest that a useful strategy for ReliefSeq may be backwards elimination or iterative removal as we have done for GWAS in Ref. [15].

### Application to Smallpox Vaccine RNA-Seq Data

In order to test the biological relevance of genes prioritized by ReliefSeq, we applied the new method to real RNA-Seq data from a recent study of 44 subjects who received the smallpox vaccine [22]. Here we use ReliefSeq to identify genes that show differences between vaccinia virus-stimulated samples versus unstimulated samples. In order to interpret the relevance of the genes prioritized by ReliefSeq, we used the Molecular Signatures Database (MSigDB) gene set enrichment resource [28] to identify significant overlap between the top 100 ReliefSeq genes in the smallpox RNA-Seq experiment and differentially expressed genes in immune system studies curated from public microarray data (c7 collection of MSigDB). Significant overlap (p = 6.25e-5, FDR q = 0.04, Table 1) was found in a set of genes that were previously found to be up-regulated in peripheral blood mononuclear cells (PBMC) stimulated with yellow fever vaccine (YF17D) in comparison with unstimulated PBMC (GSE13484) [29]. This overlap between two vaccine studies (yellow fever vaccine and smallpox vaccine) of differentially expressed genes between virus-stimulated and unstimulated cells suggests that these genes may be part of a generalized transcriptional response to immunization and viral infections. These data also demonstrate the robust nature of ReliefSeq as significant overlap was found despite the fact that the MSigDB comparison database uses microarray data and different methods for calculating differential expression. Stronger overlap is expected if compared with RNA-Seq data and ReliefSeq for gene prioritization and warrants further investigation in future analyses.

**Table 1:** Differentially expressed genes in virus-stimulated versus unstimulated cells for smallpox vaccine based on ReliefSeq that significantly overlap (p = 6.25e-5) with stimulated versus unstimulated differentially expressed genes in yellow fever vaccine in the Molecular Signatures Database (MSigDB: SE13484_UNSTIM_VS_YF17D_VACCINE_STIM_PBMC_DN).

Gene Name	Gene Description
ISG20	Interferon stimulated exonuclease gene 20 kDa
RASGRP3	RAS guanyl releasing protein 3 (calcium and DAG-regulated)
FANCA	Fanconi anemia, complementation group A
TAP2	Transporter 2, ATP-binding cassette, sub-family B (MDR/TAP)
ZBP1	Z-DNA binding protein 1

### Benchmarking

We benchmarked ReliefSeq, DESeq and edgeR on a Quad Core Intel Xeon W3520 2.66 GHz CPU, 6 GB RAM desktop. We timed each method on one of the simulated data sets, consisting of approximately 16,000 genes and 48 subjects. For a single processor, ReliefSeq for a fixed k has a runtime of about 3 seconds. ReliefSeq with gene-wise adaptive-k has a runtime of about 2 minutes. This runtime includes sweeping over all 23 possible nearest hits and misses and identifying the best k for each transcript. The runtime of DESeq is about 10 minutes and about 25 seconds for edgeR. The single processor implementation of Relief-F is quite fast for the benchmark data, which is relatively large by mRNA-Seq standards. However, if a user needs to analyze larger data sets, ReliefSeq is implemented in openMP for shared-memory parallelization to take advantage of the multiple cores available on all modern desktops. The most time consuming calculation in the Relief-F algorithm is the distance matrix between all subjects. Thus, we parallelized this matrix calculation with openMP, which uses dynamic scheduling to balance the load across the specified number of processors.

## Discussion

We have developed an extension to the Relief-F k-nearest-neighbor-based feature selection algorithm by incorporating an adaptive k that adjusts to the effect of each gene in the data set. This enables Relief-F to detect both main effects and epistatic interactions of the form described in the introduction. While the interactions associated with buffering might seem to be rare statistical effects, they have been shown to be of biological importance [7], [8], [9], [10] and should be addressed by statistical and machine learning methods for RNA-seq data. We also expect ReliefSeq to detect differential co-expression effects that manifest as statistical interactions. One expects both main effects and interaction effects that influence phenotypes to be present in RNA-seq data, and the sensitivity of Relief-F to find either effect depends on the nearest neighborhood size. We have shown on a panel of realistic simulated mRNA-Seq data the competitive performance of the gene-wise adaptive-k (gwak) ReliefSeq tool for detecting main effects and superior performance for interactions. As with differential expression, the detected effects cannot be claimed to be more than associations until further functional studies are performed.

For main effects greater than 1.5 and mean counts greater than approximately 100, all of the methods ranked these genes at the top of the list. For fold changes of 1.41, the rankings begin to deteriorate with gene means less than 1000. ReliefSeq consistently ranks buffering interactions higher than the other methods. This is not surprising given the other analytical tools were not designed to detect buffering interactions. In previous method development of Evaporative Cooling feature selection for GWAS data, we integrated importance scores from Relief-F using the traditional k = 10 nearest neighbors with those from Random Forest as a weighted sum in order to jointly utilize the ability of Relief-F to identify interactions with the ability of Random Forest to identify main effects [26]. However, due to the poor Random Forest rankings observed for simulated mRNA-Seq data, there appears to be no advantage to including Random Forest for the types of effects considered here, despite evaluating multiple implementations. Thus, in order to capture the spectrum of effects in mRNA-Seq data, we used a fast and simple procedure to find the optimum number of nearest neighbors in Relief-F, which does not appear to be at risk of false positives due to the adaptive choice of k.

Additional study is needed to determine the features of Random Forest that may explain the slightly poorer performance. Possible explanations include that Random Forests has been shown to have difficulty detecting important variables when there are only a few important variables mixed in with many unimportant variables [30], or the non-constant coefficient of variation in Negative Binomial data. Addressing these issues for Random Forest main effect analysis of RNA-Seq data will inform further improvements for gene-gene interaction analysis. For example, forest-based approaches that have been developed for identifying gene-gene interactions, like the haplotype-based approach for single nucleotide polymorphisms (SNPs) in Ref. [31], may be adapted for RNA-Seq analysis.

This tool may be used to perform feature selection prior to higher level modeling such as gene set enrichment analysis (as we did with MSigDB for real RNA-Seq data), development of predictive models, or network analysis [32]. As with other methods, ReliefSeq detects signal best for large µ and large fold changes, and performs reasonably well for fold changes as low as 1.41. When using ReliefSeq as a feature selection tool, it appears the top 40–50% of the features should be retained from the perspective of keeping informative features, in line with what others have found [33]. Since ReliefSeq utilizes group information, however, this feature selection step must be incorporated within any cross-validation loops for model building purposes [34].

For smaller sample sizes on the order of 50, it takes about 2 minutes to sweep all nearest neighbor possibilities and calculate the best k for all genes. This method makes no distributional assumptions and so will work for many other data types as well. The ReliefSeq implementation also supports GWAS and associated data formats such as the Plink format. Although these algorithm features were not used in the current study, the open-source ReliefSeq implementation supports multi-class and continuous phenotypes. For larger sample sizes, it may not be computationally feasible to sweep over all k values. For such cases, it may be necessary to set an upper k and search for the local optimum in the chosen interval. This sample size limitation is probably not relevant for current RNA-seq data sets, but it would likely become more of a concern in GWAS data where sample sizes are on the order 10^3^ or even 10^4^. Relief-F is able to incorporate interaction information when ranking the importance of a given transcript, but it does not tell you which interactions are important. In future work, we will explore exhaustive pair-wise statistical interaction models with the generalized linear model, similar to epistasis network analyses for GWAS [35].

Our manuscript has both strengths and weaknesses. Strengths include that, to our knowledge, this is the first work to consider a gene-wise adaptive choice of k in the Relief-F algorithm. This enables the algorithm to detect more types of effects. To our knowledge, this is the first interaction analysis of RNA-Seq data. Simulation parameters were determined based on data from real mRNA Seq data, and the effect sizes considered are of a magnitude observed in biological studies. We applied the method to a real RNA-Seq data set to demonstrate the identification of biologically relevant genes. Weaknesses will be addressed in future work, including the lack of a distribution for computing p-values, which limits the assessment of false positive rates. We will investigate the possibility of using the formalism developed in the current study to identify an asymptotic distribution for computing p-values, and we will investigate the computational feasibility of permutation p-values. We tested the effect of the conditionally dependent genes (interactions) in the data, but we otherwise simulated genes as independent. Future study is needed to understand the effects of correlation between genes. In addition, future development of the Relief-F method is needed to refine the estimates of k, such as parameter sweeps to minimize the cross-validation error rate and adaptive metrics.

We hope the comparison of these methods will inform future analyses of RNA-Seq data and will lead to improved characterization of transcriptome signatures for a variety of biological states that may involve complex statistical models. ReliefSeq feature selection is able to identify main effects and interactions and is applicable to data from a wide variety of distributions.

## Supporting Information

Table S1 Additional statistics on variation for the simulation results in Figure 5. The first column describes the effect of the gene in the simulated model: the fold change and negative binomial parameters. The remaining columns measure the mean, lower 5^th^ and upper 95^th^ percentile of the rank for the corresponding methods. The methods compared are Reliefseq with optimized k nearest neighbors and Relief-F with k = 10.
(XLS)

Table S2 Additional statistics on variation for the simulation results in Figure 6. The first column describes the effect of the gene in the simulated model: the fold change and negative binomial parameters. The remaining columns measure the mean, lower 5^th^ and upper 95^th^ percentile of the rank for the corresponding methods. The methods compared are Reliefseq with optimized k nearest neighbors, edgeR and DESeq.
(XLS)

Table S3 Additional statistics on variation for the simulation results in Figure 7. The first column describes the effect of the gene in the simulated model: the fold change and negative binomial parameters. The remaining columns measure the mean, lower 5^th^ and upper 95^th^ percentile of the rank for the corresponding methods. The methods compared are Relief-F with optimized k nearest neighbors is compared with Random Jungle, Random Forest (R implementation), and tuned Random Forest with optimized mtry.
(XLS)
